# Che-1 modulates the decision between cell cycle arrest and apoptosis by its binding to p53

**DOI:** 10.1038/cddis.2015.117

**Published:** 2015-05-21

**Authors:** A Desantis, T Bruno, V Catena, F De Nicola, F Goeman, S Iezzi, C Sorino, M P Gentileschi, S Germoni, V Monteleone, M Pellegrino, M Kann, P D De Meo, M Pallocca, K Höpker, F Moretti, E Mattei, H C Reinhardt, A Floridi, C Passananti, T Benzing, G Blandino, M Fanciulli

**Affiliations:** 1 Epigenetics Laboratory, Regina Elena National Cancer Institute, Via E. Chianesi 53, Rome, 00144, Italy; 2 Department of Biotechnological and Applied Clinical Sciences, University of L'Aquila, Via Vetoio Coppito 2, L'Aquila, 67100, Italy; 3 Oncogenomic Laboratory, Regina Elena National Cancer Institute, Via E. Chianesi 53, Rome, 00144, Italy; 4 SAFU, Regina Elena National Cancer Institute, Via E. Chianesi 53, Rome, 00144, Italy; 5 Institute of Cell Biology and Neurobiology, Italian National Research Council, IRCCS Fondazione Santa Lucia, Via del Fosso di Fiorano 64, Rome, 00144, Italy; 6 Department II of Internal Medicine, University Hospital of Cologne, Cologne, Germany; 7 HPC CINECA, Via dei Tizii, 6, Rome, 00185, Italy; 8 Department I of Internal Medicine, University Hospital of Cologne, Cologne, Germany; 9 Cologne Excellence Cluster on Cellular Stress Responses in Aging-Associated Diseases (CECAD), University of Cologne, Cologne, Germany; 10 IBMN-CNR, Department of Molecular Medicine, “Sapienza” University, Viale Regina Elena 291, Rome, 00161, Italy; 11 Systems Biology of Aging, University of Cologne, Cologne, Germany

## Abstract

The tumor suppressor p53 is mainly involved in the transcriptional regulation of a large number of growth-arrest- and apoptosis-related genes. However, a clear understanding of which factor/s influences the choice between these two opposing p53-dependent outcomes remains largely elusive. We have previously described that in response to DNA damage, the RNA polymerase II-binding protein Che-1/AATF transcriptionally activates p53. Here, we show that Che-1 binds directly to p53. This interaction essentially occurs in the first hours of DNA damage, whereas it is lost when cells undergo apoptosis in response to posttranscriptional modifications. Moreover, Che-1 sits in a ternary complex with p53 and the oncosuppressor Brca1. Accordingly, our analysis of genome-wide chromatin occupancy by p53 revealed that p53/Che1 interaction results in preferential transactivation of growth arrest p53 target genes over its pro-apoptotic target genes. Notably, exposure of Che-1^+/−^ mice to ionizing radiations resulted in enhanced apoptosis of thymocytes, compared with WT mice. These results confirm Che-1 as an important regulator of p53 activity and suggest Che-1 to be a promising yet attractive drug target for cancer therapy.

The DNA damage response (DDR) is a cellular defense mechanism that integrates genotoxic event detection to the activation of checkpoint pathways to arrest cells in different phases of the cell cycle to facilitate DNA repair or induce apoptosis and eliminate damaged cells.^1^ The product of the *TP53* gene plays an important role in DDR, where it works as a tumor suppressor mainly involved in the transcriptional regulation of a large number of growth-arrest- and apoptosis-related genes,^2^ and inactivation of the p53 pathway is a pivotal aspect of tumor formation in the majority of human cancers.^3^ Many factors influence the ability of p53 to determine cell fate decision. Indeed, upon genotoxic damage, p53 is rapidly subjected to a series of posttranslational modifications thought to regulate its stability and biological functions.^4^ In addition, there is a complex interplay between p53 modifications and its interaction with specific transcriptional co-factors that cooperate with p53 to induce transcriptional activation of specific targets involved in determining cellular fate.^5^

Che-1/AATF/Traube (Che-1) is a RNA polymerase II-binding protein involved in the regulation of gene transcription and cell proliferation.^6, 7, 8^ It has been shown that this protein exhibits strong antiapoptotic activity,^9, 10, 11^ and it is rapidly degraded in response to apoptotic stimuli.^12, 13^ We have previously demonstrated that in response to DNA damage, Che-1 is stabilized by ATM/Chk2 kinases and localizes to the *Tp53* promoter, increasing transcription of this gene and contributing to the increase of p53 protein levels after genotoxic stress.^14^ More recently, it has been shown that Che-1 protects cells from cell death by repressing the apoptotic arm of the p53 response,^15^ and consistent with these results, *in vivo* depletion of Che-1 is able to sensitize HCT116 tumors to antineoplastic drugs.^11, 15^

In this study, we demonstrate that in addition to sustaining *Tp53* transcription, Che-1 is a crucial/determinant component of the transcriptional complex that activates the transcription of the p53 target genes responsible of the growth arrest response. Of note, Che-1 is able to modulate p53 recruitment onto specific DNA sequences, thus promoting in this way transcriptional activation of genes involved in growth arrest and inhibiting p53 apoptotic activity. Che-1 directly interacts with p53 protein, and phosphorylation of Che-1 by ATM/Chk2 is required for such interaction, whereas Pin1-mediated modifications of p53 lead to the detachment of the two proteins. In addition, Che-1 binds the other major oncosuppressor Brca1, a component of the p53 protein complex that mediates the growth arrest response. Hence, our study uncovers an additional mechanism through which Che-1 determines the fate of the p53 pathway, offers mechanistic evidence and identifies this protein as an attractive drug target for cancer therapy.

## Results

### Che-1 binds to p53

**Figure 1:**
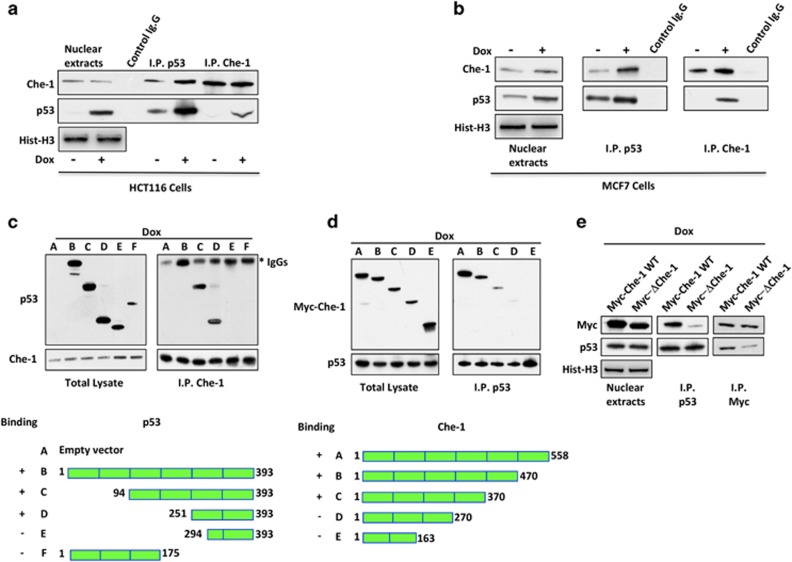
Che-1 binds to p53. (**a**) HCT116 cells were treated with 1*μ*M Dox or vehicle for 8 h. Nuclear extracts were immunoprecipitated with anti-p53 or anti-Che-1 antibodies and analyzed by western blot (WB) using the indicated antibodies (abs). (**b**) MCF7 cells were treated as shown in (**a**) and nuclear extracts were immunoprecipitated with anti-p53 antibody and analyzed by WB with the indicated abs. (**c**) HCT116 cells were transiently transfected with expression vectors containing full-length p53 or its deletion mutants and treated with 1 *μ*M Dox for 8 h. Total cell extracts (TCEs) were immunoprecipitated with anti-Che-1 antibody and analyzed by WB with the indicated abs. The bottom panel shows a schematic representation of a full-length p53 protein and its deletion mutants. (**d**) HCT116 cells were transiently transfected with expression vectors containing myc-tagged WT Che-1 or its deletion mutants and treated with 1 *μ*M Dox for 8 h. TCEs were immunoprecipitated with anti-p53 antibody and analyzed by WB with the indicated abs. The bottom panel shows a schematic representation of a full-length Che-1 protein and its deletion mutants. (**e**) HCT116 cells were transiently transfected with expression vectors containing WT Che-1 or its deletion mutant ΔChe-1 and treated with 1 *μ*M Dox for 8 h. TCEs were immunoprecipitated with anti-p53 or anti-Myc antibodies and analyzed by WB with the indicated abs

Che-1 is an important RNA polymerase II co-factor involved in DDR and p53 activation.^14, 11^ Moreover, recent data support the notion that Che-1 negatively regulates p53-driven apoptosis.^15^ All these observations prompted us to test whether Che-1 protein has the capacity for a direct and specific interaction with p53. To provide evidence in support of Che-1's association with p53 protein *in vivo*, we performed immunoprecipitations of Che-1 and p53 in the presence or absence of DNA damage. Co-precipitation of endogenous Che-1 with endogenous p53 from HCT116 (Figure 1a) or MCF7 cells (Figure 1b) demonstrated the ability of these proteins to interact and the increase of such binding in response to DNA damage.

To determine which regions of Che-1 and p53 were important for this interaction, the binding between different portions of Che-1 and p53 was tested by co-immunoprecipitations. We found that the p53 fragment containing amino acids 251–293 was required for p53/Che-1 interaction (Figure 1c). On the other hand, Che-1 mutants lacking the region spanning amino acids 270–370 did not bind endogenous p53 (Figure 1d). It is well noted that this region is involved in binding with the oncosuppressor Rb.^6^ To test whether this particular region was also required for binding Che-1 to p53, HCT116 cells were transiently transfected with Che-1 wild type (WT) and a mutant lacking the 305–323 region (ΔChe-1, upper). As shown in Figure 1e, in contrast to Che-1 WT, ΔChe-1 protein exhibited very little interaction with p53. Taken together, these results indicate that the DNA-binding domain of p53 binds the 305–323 region of Che-1.

### The DDR regulates Che-1/p53 interaction

**Figure 2:**
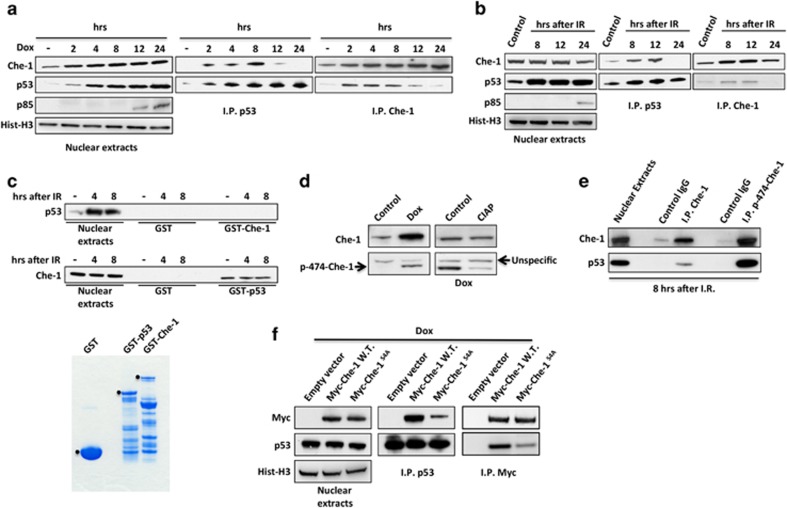
The DDR regulates Che-1/p53 interaction. (**a** and **b**) HCT116 cells were treated with 1 *μ*M Dox (**a**) or with 20 Gy IR (**b**). Nuclear extracts were immunoprecipitated with anti-p53 or anti-Che-1 antibodies and analyzed by WB with the indicated abs. (**c**) GST pull-down assay of HCT116 cells irradiated with 20 Gy performed with GST, GST-Che-1 or GST-p53 Sepharose beads. The bottom panel shows Coomassie blue staining of purified GST proteins. (**d**) TCEs from HCT116 treated where indicated with 1 *μ*M Dox and calf intestinal alkaline phosphatase (CIAP) were analyzed by WB with the indicated abs. (**e**) Nuclear extracts from IR-treated HCT116 cells were immunoprecipitated and analyzed by WB with the indicated Abs. (**f**) HCT116 cells were transiently transfected with pCS2-MT control vector, Myc-Che-1 WT or Myc-Che-1^S4A^ expression vectors and treated with 1 *μ*M Dox. Nuclear extracts were immunoprecipitated with anti-p53 or anti-Myc antibodies and analyzed by WB with the indicated abs

To determine whether the Che-1/p53 interaction is regulated during DDR, HCT116 cells were treated with the chemotherapeutic drug doxorubicin (Dox) or ionizing radiations (IR), and co-immunoprecipitations were performed using the anti-p53 or anti-Che-1 antibodies. As shown in Figures 2a and b, this interaction essentially occurred during the first hours of the DNA damage, whereas it was not observed at the later points in time when cells underwent apoptosis (as evidenced by PARP cleavage). This result suggests that DNA damage-induced posttranslational modifications might regulate the interaction of Che-1 with p53. To identify whether p53 or Che-1 need to be modified in order for them to interact with each other, we performed a reciprocal *in vitro* pull-down analysis by using bacterial recombinant proteins. From this analysis, we observed that endogenous p53 was not able to bind recombinant Che-1, whereas cellular Che-1 from IR-treated cells interacted with GST-p53 (Figure 2c), thus indicating that Che-1 modifications are required for Che-1/p53 interaction. We previously demonstrated that in response to DNA damage, ATM and Chk2 kinases phosphorylate Che-1 on specific residues and these modifications are functionally linked to DNA damage-induced G2/M checkpoint.^14^ To evaluate whether Che-1 phosphorylation is required for its binding to p53, we produced an anti-phospho-specific peptide antiserum directed against the phosphorylated Ser474 of Che-1 (Figure 2d), a Chk2 phosphorylation site.^14^ As shown in Figure 2e, the anti-p-Ser-474 Ab co-immunoprecipitated much higher levels of p53 compared with anti-Che-1 antibody, thus suggesting that Che-1 phosphorylation is involved in Che-1/p53 interaction. Consistent with these findings, a non-phosphorylable Che-1^S4A^ mutant^14^ showed an impaired ability to contact p53 when compared with Che-1 WT (Figure 2f). Taken together, these data demonstrate that Che-1 and p53 interact in response to sub-lethal DNA damage and that this binding requires Che-1 phosphorylation.

### Pin1 mediates Che-1 dissociation from p53

**Figure 3:**
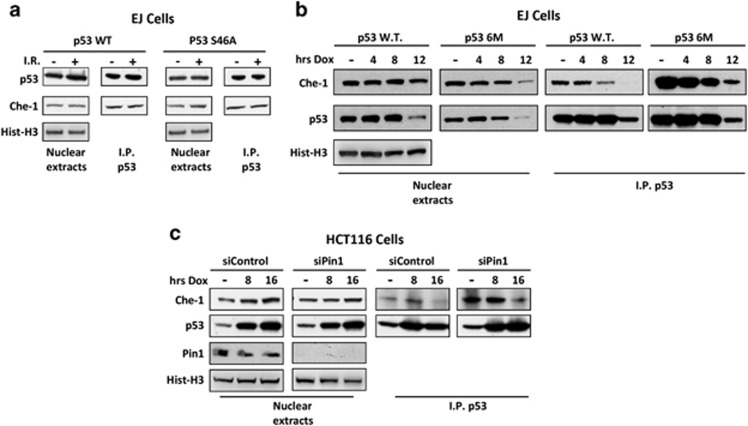
Pin1 mediates Che-1 dissociation from p53. (**a**) EJ p53^−/−^ cells were transiently transfected with WT p53 and its mutant S46A and treated with 20 Gy IR. Nuclear extracts were immunoprecipitated with anti-p53 antibody and analyzed by WB with the indicated abs. (**b**) EJ cells were transiently transfected with WT p53 and its mutant 6M that does not bind Pin1 and treated with 1 *μ*M Dox for the indicated times. Nuclear extracts treated as shown in (**a** and **c**). HCT116 cells were transiently transfected with siRNA GFP (siControl) or siRNA Pin1 (siPin1), and treated with 1 *μ*M Dox for the indicated times. Nuclear extracts treated as shown in (**a**)

We next investigated the possible mechanism by which p53 and Che-1 dissociate in response to apoptosis induction. It has been demonstrated that DNA damage-induced phosphorylations of p53 promote its binding to Pin1. This, in turn, produces a conformational change in the protein promoting cell death.^16, 17^ To assess whether these modifications might regulate Che-1 dissociation from p53, we performed co-immunoprecipitation assays using *p53*-null EJ cells overexpressing p53 WT or a p53 mutant lacking only serine 46 (S46A), a phosphorylation target involved in apoptosis induction.^18^ As shown in Figure 3a, Che-1 was able to bind both WT p53 and S46A mutant in equal measure. Conversely, a p53 mutant lacking six phosphorylation sites and unable to bind Pin1 (p53-6M)^19^ bound Che-1 more efficiently than WT p53 did upon DNA damage (Figure 3b), suggesting that additional and/or multiple phosphorylation events regulate Che-1/p53 interaction. Consistent with these results, Pin1 depletion by siRNA in HCT116 cells strongly increased p53/Che-1 interaction (Figure 3c). Altogether, these observations demonstrate that Pin1 regulates p53 dissociation from Che-1 in response to apoptosis.

### Che-1 forms a complex with p53 and Brca1

**Figure 4:**
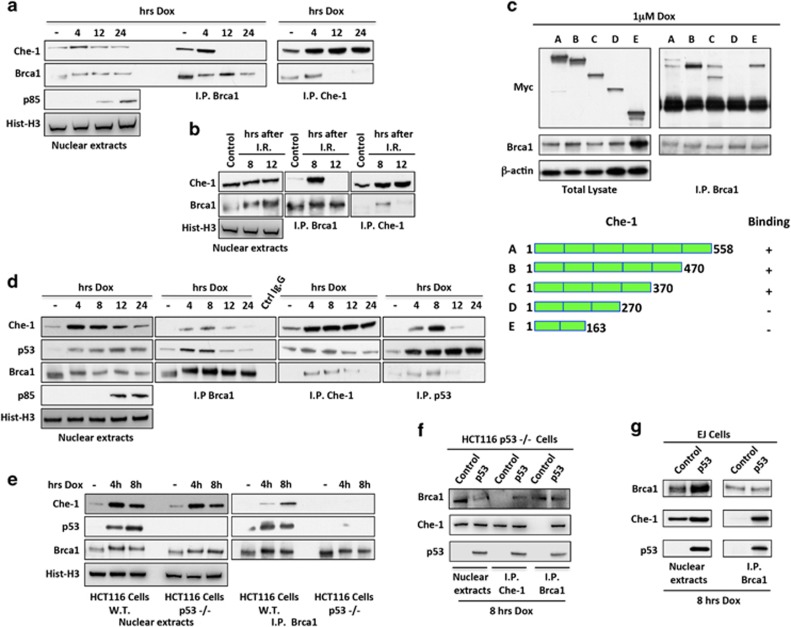
Che-1 forms a complex with p53 and Brca1. (**a** and **b**) HCT116 cells were treated with 1 *μ*M Dox (**a**) or with 20 Gy IR (**b**). Nuclear extracts were immunoprecipitated with anti-Brca1 or anti-Che-1 antibodies and analyzed by WB with the indicated abs. (**c**) HCT116 cells were transiently transfected with expression vectors containing full-length Che-1 or its deletion mutants and treated with 1 *μ*M Dox. TCEs were immunoprecipitated with anti-Brca1 antibody and analyzed by WB with the indicated abs. The bottom panel shows a schematic representation of full-length Che-1 protein and its deletion mutants. (**d**) HCT116 cells were treated with 1 *μ*M Dox. Nuclear extracts were immunoprecipitated with anti-Brca1, anti-Che-1 or anti-p53 antibodies and analyzed by WB with the indicated abs. (**e**) HCT116 WT and p53^−/−^ cells were treated with 1 *μ*M Dox. Nuclear extracts were immunoprecipitated with anti-Brca1 antibody and analyzed by WB with the indicated abs. (**f**) HCT116 p53^−/−^ cells were transiently transfected with empty vector or p53 expression vector and treated with 1 *μ*M Dox. Nuclear extracts were immunoprecipitated with anti-Brca1 or anti-Che-1 antibodies and analyzed by WB with the indicated abs. (**g**) EJ cells were transiently transfected and treated as shown in (**f**). Nuclear extracts were immunoprecipitated with anti-Brca1 antibody and analyzed by WB with the indicated abs

It has been recently demonstrated that in response to DNA damage, Che-1 modulates the cellular outcome of the p53 response.^15^ Another important factor involved in DDR, Brca1, interacts with p53 and directs its response towards growth arrest and DNA repair.^20, 21, 22^ On the basis of these observations, we investigated whether Che-1 was also able to contact Brca1. As shown in Figures 4a and b, Che-1 bound Brca1 in response to genotoxic stress with a kinetic similar to that observed in p53/Che-1 interaction. In addition, we found that Che-1 contacts Brca1 through the same region involved in the binding with p53 (Figures 4c and 1d), leading to hypothesize that Che-1, p53 and Brca1 are components of the same complex. To confirm this hypothesis, we performed immunoprecipitations by using antibodies against these proteins. We observed that all the three antibodies were able to pull down the complex at the same times in response to DNA damage, and that these interactions were lost when apoptosis occurred (Figure 4d). Next, we tested whether p53 is required for Brca1/Che-1 binding by using HCT116 cells lacking p53 expression (p53^−/−^). As shown in Figure 4e, Che-1 co-immunoprecipitated with Brca1 in HCT116 WT cells, but this interaction was not observed in cells lacking p53 expression. To further confirm these results, overexpressed p53 in HCT116 p53^−/−^ and in p53-null EJ cells, restored Brca1/Che-1 interaction (Figures 4f and g). Taken together, these findings demonstrate that in early stages of DDR, p53 forms a ternary complex containing Brca1 and Che-1, and that this complex is lost in the presence of apoptosis.

### Che-1 modulates p53 target selectivity

**Figure 5:**
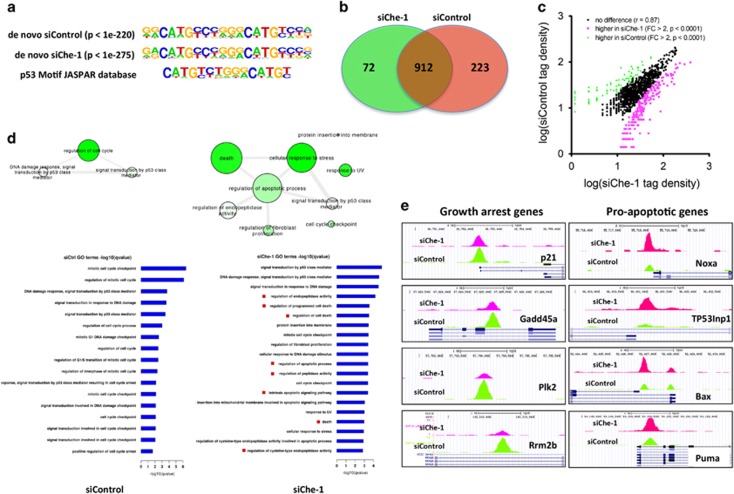
Che-1 modulates p53 target selectivity. (**a**) The p53 consensus binding motif was identified *de novo* by analyzing the sequences of peaks deriving from a p53-specific ChIP-seq with HCT116 cells transiently transfected with siRNA GFP (siControl) or siRNA Che-1 (siChe-1) and analyzed 4 h after IR treatment (20 Gy) using the program MEME. (**b**) Venn diagrams were obtained by intersection of all p53-bound DNA fragments in HCT116 cells treated as described in (**a** and **c**). Tag density correlation between the two peak sets siControl (control siRNA) and siChe-1 (Che-1 siRNA). (**d**) GO term networks constructed by REViGO. Node size indicates frequency of term in database, node color intensity reflects increasing significance as by q-values, and edge weight indicates term similarity. (**e**) ChIP-Seq data from the indicated gene promoters showing the occupancy of p53 in samples as described in (**b**)

**Figure 6:**
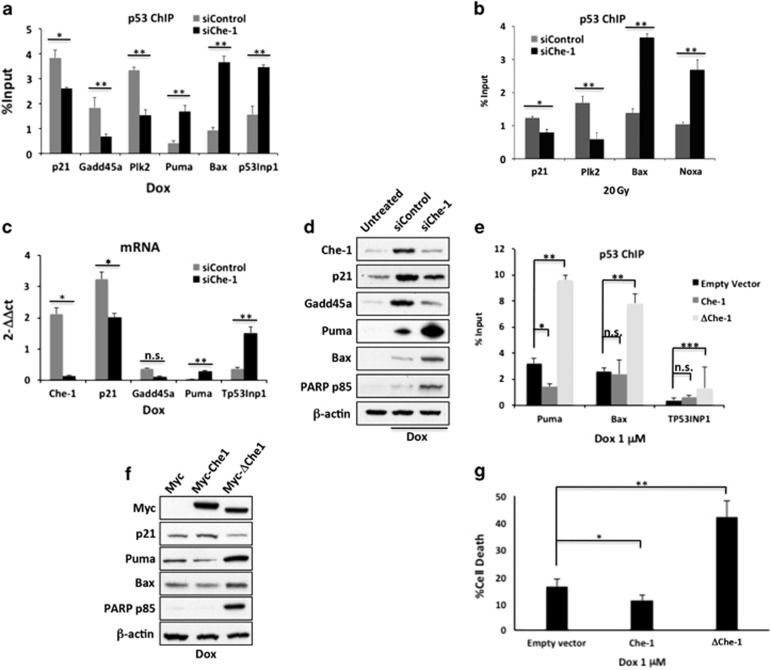
Che-1 promotes transcriptional activation of genes involved in growth arrest and inhibits p53 apoptotic activity. (**a** and **b**) Nuclear extracts from HCT116 cells transiently transfected with siRNA GFP (siControl) or siRNA Che-1 (siChe-1) and treated with 1 *μ*M Dox (**a**) or 20 Gy IR (**b**) were subjected to quantitative ChIP analysis (ChIP-qPCR) using anti-p53 antibody or control rabbit IgGs. Data are expressed as percent of input. Error bars represent the standard error of three different experiments. (**a**) *P=0.004, **P<0.0001; (**b**) *P=0.003; **P<0.0001. (**c**) Quantitative RT-PCR (qRT-PCR) for the indicated genes was performed after transient transfection of HCT116 cells with siRNA GFP (siControl) or siRNA Che-1 (siChe-1) and 1 *μ*M Dox treatment. Values were normalized to RPL19 expression. Error bars represent the standard error of three different experiments. *P≤0.0008, **P≤0.03, n.s., not significant. (**d**) WB with the indicated abs of TCEs from HCT116 cells transfected with siRNA GFP (siControl) or siRNA Che-1 (siChe-1) and treated or not with 1 *μ*M Dox. (**e**) ChIP-qPCR analysis of nuclear extracts from HCT116 cells transiently transfected with empty vector, Myc-Che-1 WT or Myc-ΔChe-1 expression vectors and treated with 1 *μ*M Dox. Data are expressed as percent of input. Error bars represent the standard error of three different experiments. *P=0.0015, **P<0.0001, ***P=0.0009, n.s., not significant. (**f**) WB with the indicated abs of TCEs from HCT116 cells transfected and treated as shown in (**e** and **g**). HCT116 cells were transfected and treated as shown in (**e**). Cell death was assayed by trypan blue staining and percentages represent trypan blue incorporating cells. Columns are average of three independent experiments and error bars indicate standard deviation. *P=0.01, **P<0.0001

To assess the impact of Che-1/p53 interaction on the transcriptional activity of p53, we performed p53 chromatin immunoprecipitation (ChIP) followed by massive parallel DNA sequencing (ChIP-Seq) in HCT116 cells treated with IR in the presence or absence of Che-1 depletion. By applying a false discovery rate of 1%, we identified 1135 peaks corresponding to p53-bound DNA in siControl cells and 984 in Che-1-depleted cells. To determine whether our peaks contained p53 response elements, we applied the *de novo* motif algorithm HOMER and found that our binding sites were indeed enriched for p53 response elements (Figure 5a). Comparison of the chromatin occupancy by p53 revealed a significant overlap between the two data sets (Figure 5b). However, an analysis of tag density showed significant differences in peaks intensity between the two samples (Figure 5c). To shed light on the biological relevance of these differences, we performed a functional annotation using the GREAT (Genomic Regions Enrichments of Annotation Tool) analysis and gene ontology biological process (GO) terms analysis of the top 400 peaks ranked by fold change. Strikingly, in the siChe-1 sample, we observed a strong enrichment of genes involved in apoptosis induction with a concomitant decrease in genes regulating cell cycle control when compared with siControl sample (Figure 5d). These results were confirmed by analyzing the extent of p53 occupancy in several genes involved in growth arrest or apoptosis. Indeed, Che-1-depleted cells exhibited a strong decrease of p53 occupancy at *p21*, *Gadd45a*, *Plk2 and Rrbm2b* promoters, with a parallel increase of this protein onto *Puma*, *Noxa*, *Bax* and *TP53Inp1* genes (Figure 5e). Subsequently, to validate the ChIP-Seq data, we performed quantitative ChIP assays of endogenous p53 from HCT116 cells treated with genotoxic agents in the presence or absence of Che-1 depletion. As shown in Figures 6a and b, under different genotoxic stresses that promote p53/Che-1 interaction, Che-1 depletion produced a strong reduction in p53 recruitment onto the promoters of cell cycle arrest genes thereby increasing the presence of p53 onto promoters of pro-apoptotic genes. Interestingly, ChIP analysis in Brca1-depleted HCT116 cells exhibited similar results (Supplementary Figure S1a). Consistent with these findings, cells with reduced Che-1 expression exhibited higher mRNA and protein levels of Puma and Bax associated with apoptosis induction, whereas p21 and Gadd45 were downregulated (Figures 6c and d). Notably, a time course analysis revealed that Che-1 depletion had an effect only when Che-1/p53 interaction took place (Supplementary Figures S1b and S1c). To better understand whether Che-1 modulates p53 target gene selection by its interaction, quantitative ChIP assays for p53 were performed using HCT116 cells treated with Dox and overexpressing Myc-tag Che-1 WT or the Myc-ΔChe-1 mutant. Whereas Che-1 WT showed little or no effect on p53 recruitment on the promoters of pro-apoptotic genes, ΔChe-1 strongly increased p53 occupancy on these promoters (Figure 6e) resulting in an increase of Bax and Puma (Figure 6f). Moreover, ΔChe-1 expression caused a significant increase of apoptosis acting thereby in a dominant-negative manner (Figures 6f and g). Taken together, these results demonstrate that Che-1/p53 binding determines cell survival upon DNA damage by regulating p53's selective target gene binding and activation.

### Che-1 modulates p53-mediated genotoxic stress *in vivo*

**Figure 7:**
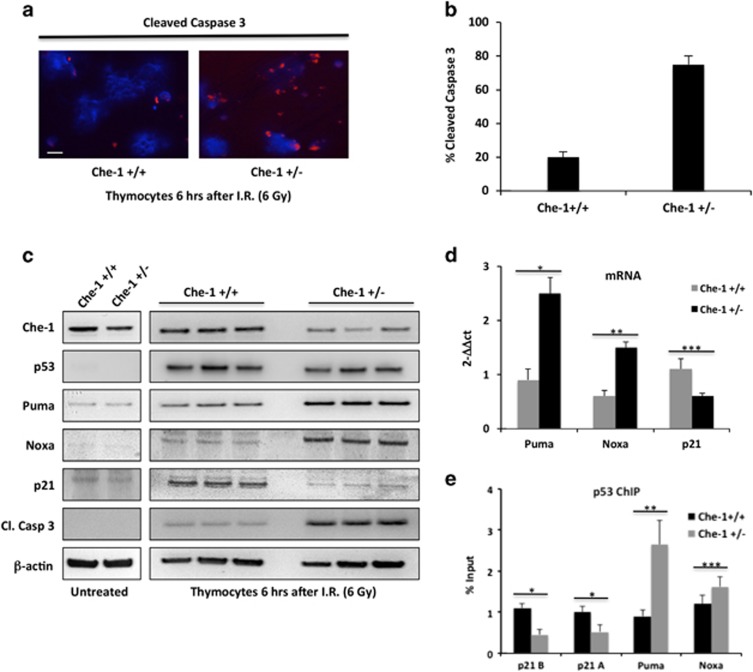
Che-1 modulates p53-mediated genotoxic stress *in vivo*. (**a**) Immunostaining for cleaved caspase-3 in thymocytes from Che-1^+/−^ and WT (Che-1^+/+^) littermates, analyzed 6 h after the mice received 6 Gy total-body irradiation. The scale bar represents 20 *μ*m. (**b**) Percentage of cleaved caspase-3-positive cells calculated from three independent experiments. Error bars are a means of three independent experiments. *P*<0.0001. (**c**) Thymocytes from three different Che-1^+/−^ mice and Che-1^+/+^ littermates irradiated or not as shown in (**a**) were analyzed by WB with the indicated abs. (**d**) qRT-PCR analysis for the indicated genes was performed from thymocytes from Che-1^+/−^ and Che-1^+/+^ littermates treated as shown in (**a**). Values were normalized to GAPDH expression. Error bars represent the standard error of three different experiments. *P=0.0002, **P=0.009, ***P=0.004. (**e**) ChIP-qPCR analysis of thymocytes from Che-1^+/−^ and Che-1^+/+^ littermates treated as shown in (**a**). Data are expressed as percent of input. Error bars represent the standard error of three different experiments. *P=0.0006, **P=0.002, ***P=0.02

Homozygous embryos that lack Che-1 die before implantation, whereas heterozygous mice bearing a null allele (Che-1^+/−^) are viable and show no developmental or tumor-prone phenotype.^7^ However, under normal conditions of adult tissue homeostasis, even in rapidly proliferating tissues, replication stress and DNA breakage are relatively low, DNA damage checkpoints are not activated,^23, 24, 25^ and the occasional DNA breaks can be recognized and repaired even in cells that carry heterozygous DDR genes. In contrast, genotoxic stress may unmask the partial DDR/repair defects of such carriers of '*conditional haploinsufficiency*', while the remaining single WT allele may no longer be adequate to fully signal enhanced DNA damage.^26, 27^ On the basis of these observations, we investigated whether Che-1^+/−^ mice were more sensitive to genotoxic insults. Che-1^+/−^ mice were produced starting from Che-1/Traube hemizygous ES cells,^7^ and were exposed together with WT mice to sub-lethal doses of IR (6 Gy). As radiation-induced apoptosis in mouse thymocytes requires p53 expression,^28, 29^ we analyzed cell apoptosis in these cells. Of note, cleaved caspase 3 resulted significantly higher in Che-1^+/−^ mice compared with WT mice (Figures 7a and b). Western blot analysis confirmed that the lower levels of Che-1 protein in Che-1^+/−^ thymocytes correlated with a concomitant increase of cleaved caspase 3 and Puma levels (Figure 7c). In agreement with these results, we also observed an increase of Puma and Noxa transcription in Che-1^+/−^ mice with a parallel decrease of p21 mRNA levels (Figure 7d). When we analyzed the presence of p53 on its target genes, we found a strong increase of p53 on Puma and Noxa promoters in Che-1^+/−^ thymocytes as compared with WT cells with a parallel decrease of p53 levels on the p21 promoters (Figure 7e), thus confirming *in vivo* that Che-1 is able to regulate p53 promoter selection.

## Discussion

The p53 tumor suppressor, which is mutated or inactivated in the majority of human cancers, functions as a master regulator of the cell response to several types of stress including DNA damage and oncogenic stimuli. To mediate these functions, the p53 protein must be activated by posttranslational modifications, which modulate p53 activity and interactions.^30^ However, the mechanism/s involved in p53 promoter selection is not yet fully understood.^31^

We had previously provided evidence that in response to DNA damage, Che-1 activates the transcription of p53 and consequently several p53 target genes.^14^ Moreover, it has been recently shown that Che-1 inhibits p53-mediated transcription of apoptotic genes.^15^ Here, we add a new mechanism by which Che-1 controls p53-mediated response: Che-1 and p53 interact and this interaction is involved in the regulation of p53 target genes selectivity, directing p53 transcription towards growth arrest genes. Che-1/p53 binding requires Che-1 phosphorylation and it occurs when cells are subjected to sub-lethal DNA damage, whereas this interaction is lost when apoptosis is activated. Finally, we show that Che-1^+/−^ heterozygous mutant mice exhibit an increased sensitivity to IR and increased p53-dependent apoptosis.

Our data demonstrate that the Che-1/p53 complex comprises the oncosuppressor Brca1, a factor known to coincide with the p53 growth arrest response. Brca1 is hypothesized to lead to chromatin decondensation inducing the initiation of transcription. Our data are in accordance with Brca1 activities that allow the recruitment of the RNA polymerase II at the p53 target promoters.

In response to DNA damage, checkpoint kinases phosphorylate Che-1 on specific residues and these modifications are functionally linked to DNA damage-induced G2/M checkpoint.^14^ Here, we show that these modifications are also required for the binding of Che-1 to p53, regulating not only the transcription of p53 but also its activities. Following DNA damage, Che-1 is phosphorylated by MK2, another important checkpoint kinase, and this modification is required for nuclear translocation of Che-1.^15^ It would be interesting to determine whether this modification is also involved in the Che-1/p53 interaction.

Our results report that the prolyl isomerase Pin1 mediates Che-1's dissociation from p53. These findings are in agreement with the notion that Pin1 binds p53 and catalyzes its phosphorylation-directed prolyl isomerization, activating the transactivation of proapoptotic p53-responsive genes.^16, 17^ Indeed, it has been described that Pin1 exerts these effects at least in part by stimulating p53 dissociation from the apoptosis inhibitor iASSP.^19^ Therefore, by modifying the conformation of p53 in response to apoptotic stress, it is reasonable to state that Pin1 can also modulate Che-1 dissociation from the tumor suppressor. p53 acetylation at lysine 120 is another important modification that mediates the decision between cell cycle arrest and apoptosis.^32, 33^ Therefore, it will be interesting to determine whether the acetylation of K120 is involved in the regulation of p53/Che-1 binding.

**Figure 8:**
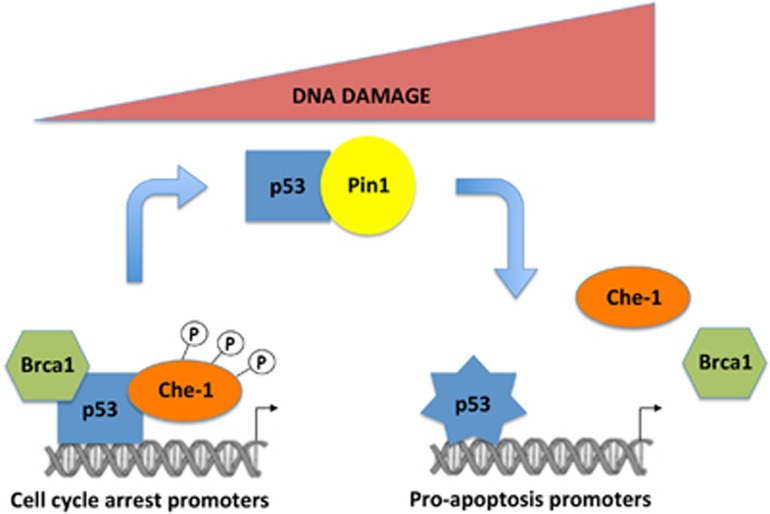
Model to explain the effects of Che-1/p53 binding. In the first hours of DNA damage, phosphorylated Che-1 sits in a ternary complex with p53 and Brca1, directing p53 transcription towards growth arrest genes. When cells undergo apoptosis, Pin1 produces p53 modifications resulting in the disassembly of the complex and p53 is free to be recruited to the pro-apoptotic genes

We show that Che-1 is present in a ternary complex with p53 and Brca1 and that p53 is required for these interactions. Consistent with these observations, p53 binds these proteins through different domains (Figure 1c).^20^ Notably, our data indicate that the induction of a p53-mediated apoptotic response is correlated with a reduction of its binding to Che-1 and Brca1. These findings are in agreement with the role of these proteins in directing p53-mediated cellular outcomes towards cell cycle arrest.^15, 21, 22^ Therefore, our results enable us to propose a model in which the binding of p53 with Che-1 and Brca1 is required for recruiting this complex onto cell cycle arrest genes However, when these interactions are lost, p53 exhibits a stronger affinity for the promoters of pro-apoptotic genes (Figure 8).

Our data also support the model that sees apoptosis as the final outcome initiated when cells become aware that DNA damage is not repairable. At this point, the protein complex Che-1/p53/Brca1 is disassembled and p53 is free to be recruited to the pro-apoptotic genes. It would be interesting to know whether the phosphorylations that regulate this process are able to release the repressive activity of Che-1 onto the apoptotic promoters.

Using hemizygous Che-1^+/−^ mice, we carried out an analysis of Che-1 function in response to irradiation under *in vivo* conditions. We found that exposure to IR results in increased apoptosis in Che-1^+/−^ mice as compared with WT littermates. Moreover, thymocytes from Che-1^+/−^ mice exhibited higher levels of p53 on pro-apoptotic gene promoters. Together, these results indicate that Che-1 controls p53 specificity in a haplo-insufficient manner repressing transcription of pro-apoptotic genes both *in vitro* and *in vivo*. Therefore, our findings reinforce the notion that Che-1 is a valid therapeutic target to increase the efficacy of antineoplastic drugs.

## Materials and Methods

### Cell culture and transfections

HCT116 WT, HCT116 p53^−/−^, EJ and MCF7 cell lines were cultured in D-Mem High glucose or RPMI1640 with 10% fetal calf serum, respectively. Transfections were carried out by Lipofectamine 2000 (Life Technologies, Carlsbad, CA, USA) following the manufacturer's instructions. Dox was purchased from Sigma (St. Louis, MO, USA). Calf intestinal alkaline phosphatase was purchased from Life Technologies.

### Plasmids and antibodies

Myc-tagged Che-1 and its mutants Myc-Che-1^S4A^ ^(ref. 14)^ and Myc-ΔChe-1^(ref. 8)^ were already described. WT p53 and its p53 mutants S46A, p53-6M, were kindly provided by Prof. G. Del Sal. Glutathione-S-transferase (GST) fusion proteins were already described.^14^ The following rabbit polyclonal antibodies were used: anti- Che-1,^6^ Brca1 (Millipore-Upstate, Darmstadt, Germany), PARP-1 p85 fragment (Promega, Madison, WI, USA), p53 Ab-7, Puma, Noxa, Gadd45a (Calbiochem, Darmstat, Germany), p21 (Santa Cruz, Dallas, TX, USA), Cleaved caspase-3 (Cell Signaling, Danvers, MA, USA), Histone H3 (Abcam, Cambridge, UK). Mouse monoclonal antibodies anti- p53 (DO1), *β*-actin (Sigma), Myc 9/10 and Pin1 (Life technologies) were also used. Phosphorylation site-specific anti-p-Che1-474 antibody was produced and purified by the company Phospho-Solution (Aurora, CO, USA). Secondary antibodies used were goat anti-mouse and goat anti-rabbit, conjugated to horseradish peroxidase (Bio-Rad, Hercules, CA, USA). Immunostained bands were detected by the chemiluminescent method (Amersham, Uppsala, Sweden).

### Western blot analysis and co-immunoprecipitations

Cell extracts were prepared as previously described.^14^ Solubilized proteins (25 *μ*g) were resolved on Mops NuPAGE precast 4–12% gels (Life Technologies). Precleared nuclear extracts were incubated with protein A/G-sepharose (Pierce, Carlsbad, CA, USA) in lysis buffer containing 0.05% BSA and specific antibody, under constant shaking at 4 °C for 2 h. After incubation, Sepharose beads-bound immuno-complexes were rinsed with lysis buffer and eluted in SDS sample buffer for western blot analysis.

### RNA isolation and qRT-PCR analysis

Total RNA from cells being harvested 36 h after transfection or from mouse thymocytes was isolated using TRIZOL reagent (Life Technologies) in accordance with the manufacturer's instructions, and the first-strand cDNA was synthesized with random primers and SuperScript II reverse transcriptase (Life Technologies). The cDNA was used for quantitative real-time PCR (qRT-PCR) experiments carried out in the 7500 Fast Real Time PCR System (Applied Biosystem, Carlsbad, CA, USA) using SYBR GREEN PCR Master Mix (Applied Biosystem). ΔΔCt values were normalized with those obtained from the amplification of the endogenous RPL19 gene or GAPDH (in mice).

The following primers were used:

mouse Puma forward: 5′-CGGCGGAGACAAGAAGAG-3′

mouse Puma reverse: 5′-CTCCAGGATCCCTGGGTAAG-3′

mouse Noxa forward: 5′-CCACCTGAGTTCGCAGCTCAA-3′

mouse Noxa reverse: 5′-GTTGAGCACACTCGTCCTTCAA-3′

mouse p21 forward: 5′-GCAGACCAGCCTGACAGATT-3′

mouse p21 reverse: 5′-CCTGACCCACAGCAGAAGAG-3′

mouse Gapdh forward: 5′-AAGGGCTCATGACCACAGTC-3′

mouse Gapdh reverse: 5′-GGATGCAGGGATGATGTTCT-3′

human RPL19 forward: 5′-CGGAAGGGCAGGCACAT-3′

human RPL19 reverse: 5′-GGCGCAAAATCCTCATTCTC-3′

human Puma forward: 5′-GACGACCTCAACGCACAGTA-3′

human Puma reverse: 5′-CTGGGTAAGGGCAGGAGTC-3′

human Gadd45a forward: 5′-CCGAAAGGATGGATAAGGTGGG-3′

human Gadd45a reverse: 5′-CATGTAGCGACTTTCCCGGCAA-3′

human Che1 forward: 5′-AGCGCTTTGCCGACTTTACA-3′

human Che1 reverse: 5′-GCTTGGTCTGTGTCCTTCGAA-3′

human TP53INP1 forward: 5′-ATGTCCAATGGAGGAGAGCTGG-3′

human TP53INP1 reverse: 5′-TATCCACTGGGAAGGGCGAAAG-3′

human p21 forward: 5′-CCTGGCACCTCACCTGCTCTGCTG-3′

human p21 reverse: 5′-GCAGAAGATGTAGAGCGGGCCTTTG-3′

### ChIP sequencing

ChIPs from HCT116 cells treated with IR (8 h after irradiation) in the presence or absence of Che-1 depletion were performed as previously described^8^ by using rabbit polyclonal anti-p53 antibody. The quantity of the immunoprecipitated material was determined by Qubit 2.0 fluorometer (Life Technologies). Ten nanograms of the immunoprecipitated chromatin were used to prepare the libraries for sequencing following the manufacturer's instructions including DNA end repairing, adaptor ligation and amplification (Illumina, San Diego, CA, USA). Fragments of about 100–180 bp (without linkers) were isolated from agarose gel and used for sequencing using the Illumina GA IIx. (36 bp, 21–26 Mio quality-filtered and uniquely aligned reads per sample).

### Data analysis

The fastq files generated by the Illumina pipeline were mapped to the human genome using Bowtie allowing up to 2 mismatches. Only uniquely mapped reads were kept. An input control library served as negative control. Peak calling was performed with MACS2^34^ using default parameters for model-building (bandwidth 300) and a q-value cutoff of 0.001. *De novo* motif enrichment analysis was carried out with HOMER software on a repeat-masked hg18 genome.^35^ GO terms enriched among p53 target genes were identified using the GREAT^36^ algorithm with basal plus extension rule settings (5 kbp upstream, 1 kbp downstream, 100 kbp extension; curated domains included) and false discovery rate cutoff values of 0.01 in both, binomial and hypergeometric tests. GO terms passing the false discovery rate threshold were summarized in networks using REVIGO^37^ with a SimRel similarity cutoff of 0.5. Motif and GO enrichment analyses were restricted to a set of 400 peaks with the highest fold-enrichment values in each dataset.

### Chromatin immunoprecipitation assays

ChIP assays were performed as previously described^8^ by using anti-p53 antibody from cells treated with Dox for 8 h or IR (8 h after irradiation). Immunoprecipitations with non-specific immunoglobulins (Santa Cruz) were performed as negative control. For quantitative ChIP analysis (ChIP-qPCR), 1 *μ*l of purified DNA was used for amplification on an Applied Biosystems 7500 Fast Real Time PCR system (with Applied Biosystem SYBR GREEN).

The following primers were used:

human Plk2 forward: 5′-GCCATTAGAGAGGAGAAAGGG-3′

human Plk2 reverse: 5′-CCCCTGGGCTTATGAATAAAG-3′

human Puma forward: 5′-AAGTCAGGACTTGCAGGCGCG-3′

humna Puma reverse: 5′-TGGGTCCCAGTCAGTGTGTGT-3′

human Gadd45a forward: 5′-TGGTGACGGTAAGGGACTGGG-3′

human Gadd45a reverse: 5′-CCCCAGCATGCTTAGCTTAGA-3′

human TP53INP1 forward: 5′-GAGGTTGTCACCAACGCACGT-3′

human TP53INP1 reverse: 5′-TGAGGGAGAGATCCACCTCTG-3′

human Bax forward: 5′-TCCCGGCTCTCTGATCCCCG-3′

human Bax reverse: 5′-GGCTAGGGGAACGCTATATGC-3′

human p21 forward: 5′-CCTGCTTCCCAGGAACAGGCT-3′

human p21 reverse: 5′-CAGGCTAAGGTTTACCTGGGG-3′

human Noxa forward: 5′-CGCTGACGACGTCCCAGCGTTT-3′

human Noxa reverse: 5′-CGAAGACGGCGTTATGGGAGC-3′

mouse p21 forward, 5' site: 5′-GAG ACC AGCAGCAAAATCG-3′

mouse p21 reverse, 5' site: 5′-CAGCCCCACCTCTTCAATTC-3′

mouse p21 forward, 3' site: 5′-TCACAGAAGAGGAGGCCTGT-3′

mouse p21 reverse, 3' site: 5′-TCCTGCTTTGGAGAAGCTGT-3′

mouse Puma forward: 5′-CACCCTAGGTCTGGGCTGT-3′

mouse Puma reverse: 5′-AAGTCGGGGCTTGCAGTC-3′

mouse Noxa forward: 5′-AAGCAATTTGGGGGTTGAG-3′

mouse Noxa reverse: 5′-GACGTCATGTGACGACATCC-3′

### Immunofluorescence assays

Immunofluorescence analyses of cleaved caspase-3 on mice thymocytes were performed using cytospin preparations of cells mounted on glass slides using a Thermo Shandon cytospin 2 (Thermo Fisher Scientific, Carlsbad, CA, USA). Briefly, cells were fixed in 4% formaldehyde for 15 min and then permeabilized with 0.1% Triton X100 in phosphate-buffered saline for 5 min. Primary antibody was used for immunostaining, followed by Alexa-Fluor-594-conjugated anti-rabbit IgG (Life Technologies). Nuclei were visualized by staining with 1 *μ*g/ml Hoechst dye 33258 (Sigma). As a control, the primary antibodies were omitted (data not shown). Immunofluorescence analysis was performed using the microscope Axioskop 2 plus and fluorescence signals were analysed by recording images using a CCD camera (Zeiss, Oberkochen, Germany).

### siRNA

The 22-nucleotide siRNA duplexes corresponded to nucleotides 1062–1083 (siChe-1), of human Che-1 sequence, and to nucleotides 122–143 of the negative control green fluorescent protein (GFP). Sequences were synthesized *in vitro* by using the Silencer siRNA construction kit (Ambion, Carlsbad, CA, USA) following the manufacturer's instruction. RNA interference was performed as previously described.^8^ siRNA-mediated interference experiments of Pin1 and Brca1 expression were performed by transfecting a specific pool of double-stranded RNA oligonucleotides (Stealth, Life Technologies) using Lipofectamine 2000 (Life Technologies).

### *In vivo* experiments

Age-paired Che1^+/−^ and Che-1^+/+^ control mice received a sublethal dose of 6 Gy whole-body γ-irradiation using a Cesium-137 irradiator. After 6 h, mice were killed by CO_2_, the thymus was removed and thymocytes were isolated by filtration using a 100 *μ*m mesh membrane (Millipore). All procedures involving animals and their care were conducted in accordance with institutional guidelines and regulations.

### Accession number

All ChIP-seq raw data have been deposited at the National Center for Biotechnology Information (NCBI) Gene Expression Omnibus (http://www.ncbi.nlm.nih.gov/geo) under the accession number GSE60267 (submitter M Fanciulli).

### Statistical analysis

Statistical analysis was performed by using the Student two-tailed *t*-test to compare *in vitro* experiments. All statistical tests were carried out using GraphPad Prism version 5.0 for Windows, Graphpad Software, San Diego, CA, USA (www.graphpad.com). Probability value of <0.05 was considered statistically significant.

## Abbreviations

Che-1: Che-1/AATF
DDR: DNA damage response
Dox: Doxorubicin
IR: Ionizing radiations
ChIP: Chromatin immunoprecipitation
GST: Glutathione-S-transferase
GFP: Green fluorescent protein
