# Supplementary figures and images for: Che-1 modulates the decision between cell cycle arrest and apoptosis by its binding to p53
Source: Cell Death Dis. 2015 May 21;6(5):e1764–. doi: 10.1038/cddis.2015.117 (PMC4669697; doi:10.1038/cddis.2015.117)

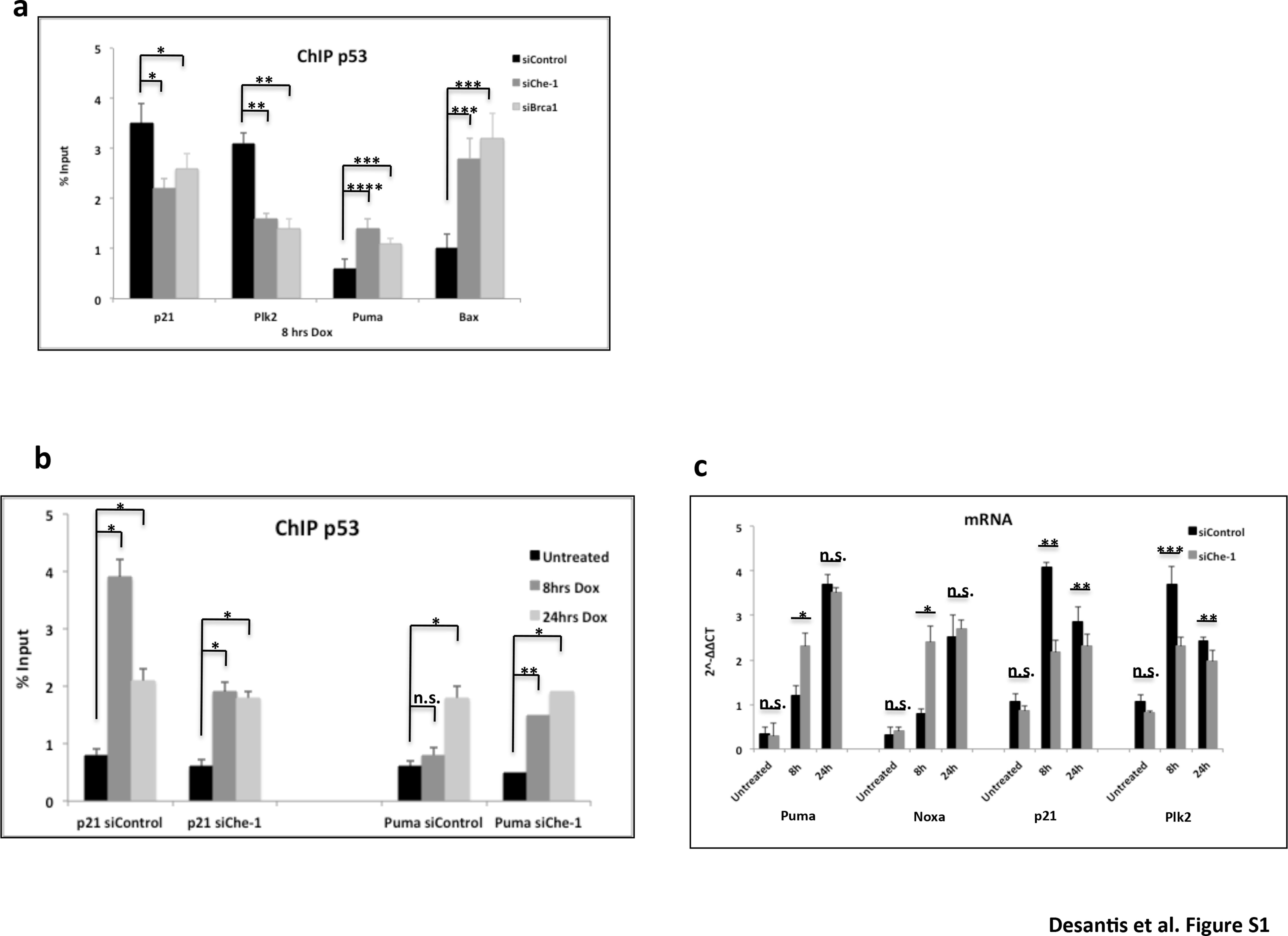

Supplement: Supplementary Figure S1 [file cddis2015117x1.tif]
