# Supplementary material for: Che-1 modulates the decision between cell cycle arrest and apoptosis by its binding to p53
Source: Cell Death Dis. 2015 May 21;6(5):e1764–. doi: 10.1038/cddis.2015.117 (PMC4669697; doi:10.1038/cddis.2015.117)
Supplement: Supplementary Information [file cddis2015117x2.doc]

***Figure S1 (related to Figure 6): Che-1 promotes transcriptional activation of genes involved in growth arrest and inhibits p53 apoptotic activity.***

**a:** Nuclear extracts from HCT116 cells transiently transfected with siRNA GFP (siControl), siRNA Che-1 (siChe-1), or siRNA Brca1 (siBrca1) and treated with 1 μM Dox, were subjected to quantitative ChIP-qPCR using anti-p53 antibody or control rabbit IgGs. Data are expressed as percent of input. Error bars represent the standard error of three different experiments. (*p=0.002, **p≤0.0001, ***p=0.008, ****p=0.03). **b:** Nuclear extracts from HCT116 cells transiently transfected with siRNA GFP (siControl) or siRNA Che-1 (siChe-1), and treated with 1 μM Dox for the indicated times, were subjected to quantitative ChIP-qPCR using anti-p53 antibody or control rabbit IgGs. Data are expressed as percent of input. Error bars represent the standard error of three different experiments. (*p≤0.0008, **p=0.005, n.s.: not significant). **c:** Quantitative RT-PCR (qRT-PCR) for the indicated genes was performed after transient transfection of HCT116 cells with siRNA GFP (siControl) or siRNA Che-1 (siChe-1) and 1 μM Dox treatment for the indicated times. Values were normalized to RPL19 expression. Error bars represent the standard error of three different experiments. *p≤0.0003, ** p≤0.01, ***p≤0.04, n.s.: not significant.
